# ‘They are my future’: childbearing desires and motivations among women with disabilities in Ghana - implications for reproductive healthcare

**DOI:** 10.1186/s12978-020-01000-y

**Published:** 2020-10-06

**Authors:** John Kuumuori Ganle, Rebecca Racheal Apolot, Tafadzwa Rugoho, Joshua Sumankuuro

**Affiliations:** 1 Department of Population, Family and Reproductive Health, School of Public Health, University of Ghana, Legon, P.O. Box LG 13, Accra, Ghana; 2 Stellenbosch Institute for Advanced Study (STIAS), Wallenberg Research Centre at Stellenbosch University, Stellenbosch, 7600 South Africa; 3 Department of Health Policy, Planning and Management, Makerere University School of Public Health, Makerere University, Kampala, Uganda; 4 Department of Development Studies, Great Zimbabwe University, Masvingo, Zimbabwe; 5 School of Community Health, Faculty of Science, Charles Sturt University, Bathurst, New South Wales Australia

**Keywords:** Women with disabilities, Sexuality, Childbearing, Desire, Intension, Motivation, Motherhood, Ghana

## Abstract

**Background:**

Previous research has highlighted widespread public mis/perceptions that portray women with disabilities (WWDs) as asexual, less likely to marry, and often not interested in childbearing. However, evidence from high-income settings shows that many WWDs are sexually active and do have or want to have children. Notwithstanding this, very few studies have focused on understanding childbearing desires and motivations among WWDs in low-income settings. This qualitative research explored childbearing desires and motivations among WWDs in Ghana.

**Methods:**

A cross-sectional qualitative study was conducted with WWDs aged 18–49 years in Northern Ghana. The distribution of participants by disability types were as follows: physical disability/impairment (*n* = 37); visual impairment (*n* = 11); speech and hearing impairment (*n* = 14); epilepsy (*n* = ten); and albinism (*n* = five). A pre-tested open-ended thematic topic guide was designed and used to conduct in-depth interviews. Interviews were tape-recorded and later transcribed for analysis. Transcripts were coded using QSR NVivo 11 software. Thematic content analysis techniques were used to analyse and present the data.

**Results:**

Nearly all the WWDs interviewed were sexually active, desiring to have children, and intended to have as many children as they could support. Strong desire to experience the joy of motherhood; fear of social insecurity; fear of old age economic insecurity; desire to challenge stigma and negative stereotypes about disability, sexuality and motherhood; and desire for self-actualisation, were key motivations for childbearing.

**Conclusion:**

Our findings challenge existing negative public perceptions about the status of WWDs in relation to sexuality, childbearing and motherhood. More importantly, our findings suggest that if the Sustainable Development Goals related to universal access to sexual and reproductive healthcare are to be attained, WWDs must be targeted with quality sexual and reproductive healthcare information and services.

## Plain English summary

Evidence from high-income settings shows that many women with disabilities do desire to get pregnant and give birth to their own children. In low-income countries however, there is poor understanding about what motivates the desire to have children among women with disabilities. This poor understanding could hamper provision of appropriate information, counselling and essential services to help women with disabilities properly plan and time their pregnancies. We conducted individual interviews with 77 women with different disabilities in Ghana to understand what their motivations for childbearing were. We found that many of these women wanted to have as many children as they could care for. Their motivations for wanting to have children were categorised into five. First, these women simply wanted to enjoy the experience of being a mother. Second, many of them wanted to have children because they were afraid of losing their marriage and/ or respect from family and community members. Third, many feared that if they did not have children, there will be no one to care for them (women) in old age. Fourth, others wanted to have children to show that having a disability did not mean that they could not have children and be mothers. Finally, many said they wanted to give birth because doing so made them feel empowered as valuable members of their families and communities. We recommend that women with disabilities should also be targeted with quality essential sexual and reproductive healthcare information, counselling and services to support them plan and time their pregnancies appropriately.

## Background

Disability may be defined as the consequence of an impairment that could be physical, cognitive, mental, sensory, emotional, developmental, or some combination of these that interacts with the natural or built environment to restrict an individual’s ability to participate in what is considered normal in their everyday society [1]. Globally, 15% of the human population is made up of persons with disabilities (PWDs) [1]. This rate varies in the general population and in specific groups. For instance, among women in their childbearing years (15–49 years), approximately 11% has a disability [2]. In the US, 15.7% of non-institutionalized women aged 18–44 report serious functional limitations related to vision, cognition, mobility, self-care, and/ or independent living [3]. In Ghana, disability prevalence among women and men is 10.6 and 6.2% respectively [4, 5].

Considerable research has highlighted the fact that even though the United Nations’ Convention on the Rights of Persons with Disabilities (UNCRPWDs) has brought improvements in many aspects of PWDs’ lives [2], they are still one of the most marginalized and socially excluded groups in many countries, including Ghana [6–8]. In particular, women with disabilities (WWDs) are more likely to be poorer and have lower social and economic status than their counterparts who have no disability [6–8]. In Ghana, WWDs face similar difficult circumstances. For instance, among PWDs aged 6–24 years, 22% of females have never attended school compared to 19% of males [4]. Similarly, whereas 61% of males with disabilities aged 15 years and above are in some kind of employment, only 54% of women with disabilities aged 15 years or more are in employment [4]. In addition to having lower opportunities for education and employment, WWDs also face other social and gender-based discrimination. For instance, in a highly gendered society like Ghana, women with physical disabilities often suffer more than men because in patriarchal culture, women tend to be judged harshly more by their bodies than do men [8]. Similarly, WWDs are more likely to be accused of witchcraft and/ or suffer more gender-based violence, including sexual violence than men with disabilities [8].

On matters of sexuality and reproductive health, research suggests that WWDs have largely been ignored [8, 9]. As Kallianes & Rubenfeld note, both the women’s and disability rights’ movements have paid scant attention to the concerns of WWDs, especially involving sexuality, reproductive freedom and mothering [10]. Indeed, despite Ghana being a signatory to the UNCRPWDs, and despite enacting the Persons with Disability Act (Act 715) in 2006, one recent review on disability in sexual and reproductive health policies and research in Ghana concluded that PWDs have received little attention [11]. This neglect has been widely reported in a number of other low-income settings [8, 12–21]. Reasons for such neglect include the fact that PWDs are often thought to be asexual, less likely to marry or to want to have children, negative social attitudes and cultural assumptions, and healthcare providers’ limited knowledge about disability [8, 12–17, 22].

With specific reference to fertility desires and motivations for childbearing, evidence suggests that many WWDs desire and are able to have children [23, 24]. Fertility rates are, in fact, similar among those with and without disability [23, 24]. For instance, a large study of 10,718 women who responded to the US National Survey of Family Growth from 2006 to 2010 showed that those with and without disability have similar attitudes, desires, and intentions towards childbearing and motherhood [23]. WWDs were about as likely to want a baby (61%) as women without disabilities (60%) [23].

In low-income settings, a few area-based qualitative studies have also highlighted the fact that many WWDs are sexually active and do have or want to have children [8, 21, 25–27]. What remains relatively underexplored is WWDs’ motivations for childbearing. As some recent studies point out, very few studies have focused on understanding childbearing desires and motivations among WWDs, especially in low-income settings [2, 23, 27]. Similarly, while recent research highlights the role of attitudes, subjective norms and perceived behavioural control in the formation of fertility intentions in the general population [27], there is no specific focus on PWDs. Deepening understanding on the motivations for childbearing among WWDs could be essential not just for fertility regulation but also for planning and delivering high-quality reproductive healthcare to WWDs [23]. This qualitative research explored childbearing desires and motivations among women with physical disability/impairment, visual impairment, speech and hearing impairment, epilepsy, and albinism in Ghana.

## Conceptual framework

In the social and behavioural sciences, many studies have explored such questions as what motivates people to bear children [28–36]. From these studies, a number of explanatory frameworks have emerged, including psychological theories of reasoned action [37], the values and dis-values that children have for parents [38], microeconomic or demand-for-children models of fertility [39], the Traits-Desires-Intentions-Behaviour model of fertility [29], social norms and roles theories of fertility desire [10], the sexual drive and desire for physical intimacy framework [29], and the maternal drive framework (i.e. motherhood is often reflected as a typical part of every woman’s female identity) [40]. Among the different frameworks reviewed prior to this study, Miller’s [29] fitted our study as it addresses the issues of childbearing motivation and desire in an encompassing manner.

In ‘Childbearing motivations, desires, and intentions: a theoretical framework’**,** Miller [29] argues that desires are psychological states that represent what someone wishes for or wants. In other words, desires are wishes or feelings about possible goals or objectives, and are influenced primarily by factors internal to the individual. They do not usually lead directly to action until they are converted, through the appraisal of reality and arrival at a decision, into intentions [29]. As shown later in this paper, some WWDs expressed a desire to have their own children. However, this desire is mediated by personal circumstances and realities, including whether they will be able to care for a child. Linked to desire is motivation. Motivations in the context of fertility and childbearing represent the disposition of people to react favourably or unfavourably to childbearing and its various aspects [29]. Thus, motivation and desire are connected in so far as particular motivations may trigger a desire to have a child. For example, and as shown later in this paper, some WWDs desired to have their own children precisely because they are motivated by fear of social insecurity as well as old age economic insecurity. For Miller [28, 29], motivation has both energizing and directional aspects: the energizing component produces a readiness to act, while the directional aspect provides specific direction to any action. Miller [28, 29] further argues that motivations are generally latent in the individual (i.e. at any given time they may be influencing neither behaviour nor a part of the individual’s consciousness). However, under certain conditions that are specific for each type of motivation, they may be aroused, at which time they begin to affect fertility behaviour [28, 29]. Thus, childbearing motivations may undergo several changes before they are translated into behaviour and ultimately into fertility events [29]. In this study, we used Miller’s [29] conceptual framework to help us understand the childbearing desires and motivations of WWDs in Ghana.

## Methods

### Study design

A cross-sectional qualitative study was conducted. The design formed part of a larger multi-methods study that was conducted to examine the sexual, reproductive and maternal and child healthcare needs, behaviours and challenges of WWDs aged 18–49 years in Ghana between January 2018 and June 2019.

### Study context

Empirical research was conducted in three districts (Central Gonja, Savelugu-Nanton, and Bunkpurugu-Yunyoo) in the Northern region of Ghana. We selected these districts because they had the highest disability rates in the region: Bunkprugu-Yunyoo (5.4%), Savelugu-Nanton (4.6%), and Central Gonja district (3.6%) [41]. At the time of this research, the northern region was the largest in Ghana in terms of land area (approx. 70,383 km^2^). Its population was approximately 2,779,877, representing about 10.2% of the national total population [41]. Females constituted 50.3% of the total regional population, and population growth rate was 2.9 per annum [41]. About 60% of the population identified themselves as Muslims [41].

In terms of fertility and reproduction, the region is very pro-natalist, and had a total fertility rate of 6.6 children per woman as compared to the national average of 4.2 [42]. Child-bearing begins as early as 12 years, with mean number of children ever born to a woman being 5.6 [41]. Some 54.3% of the population aged 12 years and above were married [41]. Modern contraceptive use (17%) in the region was the lowest in the country [42].

Patriarchy and patrilineal descent ideologies shaped everyday social relationships in most communities in the region. Polygyny was a common form of marriage, affecting 42 and 27% of women and men respectively [41, 43]. In many communities, husbands often made decisions about childbearing and family planning as well as decisions on when to have another child and the number of children to have [44, 45].

### Participants

Adult women aged between 18 and 49 years who had a disability either from birth or before they turned age 18 constituted the participants. We focused on disability before adulthood in order to better understand how this early experience of disability affected or would affect sexual health, reproductive and childbearing decisions. In total, 77 WWDs were included in the study. These were spread across five impairment/disability types: epilepsy (*n* = ten), physical disability/impairment (e.g. neuromuscular diseases, spina bifida, spinal cord injury, limbs amputation, muscular dystrophy, and polio-related injuries; *n* = 37), visual impairment (full blindness; *n* = 11), speech and hearing impairment (*n* = 14), and albinism (*n* = five).

### Participant selection and recruitment

A combination of purposive and snowball sampling techniques was used. The procedure for contacting and recruiting WWDs involved a number of steps. We contacted leadership of the Ghana Federation of Disability Organisations (GFD) at the regional capital (i.e. Tamale). The GFD is an umbrella civil society organization, comprising disability-specific groups in Ghana. We explained the purpose of our research and solicited support to recruit eligible participants. From this initial engagement, the research team was introduced to district-level leaders and representatives of various disability organisations in the three study districts. At the district levels, the GFD and local district assemblies had databases of PWDs. Following engagements with district-level leaders and representatives of various disability organisations, we were granted access to these databases. From these databases, we identified potentially suitable participants for inclusion in our study. The research team and Community-based Surveillance Volunteers (CBSVs) visited potentially eligible WWDs in their respective communities, where the purpose of the study and sampling procedures were explained.

Our initial engagement with potential participants allowed them to ask questions about the study, which the research team gladly answered. Each potential participant was given 1 week (from the date of this initial meeting) to decide whether to participate in the study. This was particularly important for those who were married, cohabiting or depended on their parents or other family members. Each participant was re-contacted by the volunteers after the one-week period. When the decision was in favour of participation, interview date, venue, time and language were agreed. We initially approached 75 eligible participants. Eight of them refused participation mainly due to husband’s/partner’s/guardian’s disapproval. However, a number of those who refused participation suggested and directed us to a total of ten other potential participants, most of whom were not registered with either their local district assembly or the GFD in the district. Our final sample was 77 WWDs.

### Data collection methods

We collected data using in-depth interviews (IDIs). Many (*n* = 52) of the interviews were conducted in the local dialects: *Gonja* and *Twi* in Central Gonja district; *Dagbaani* in Savelugu-Nanton district; and *Moar* and *Komba* in Bunkpurugu-Yunyoo district. Four research assistants (two females with disabilities; one male with disability; and one male without disability) were trained to conduct the interviews in the local dialects. We also recruited and trained one female research assistant from the Ghana Association of the Deaf to conduct interviews using Sign Language with 14 participants who had speech/hearing impairments. All our research assistants were teachers with either Teacher Training College qualification or a university first degree. A few (*n* = 11) of the interviews were however conducted in English. Except five interviews, all participants gave permission for the interviews to be recorded with an audio-tape recorder. Hand-written field notes were also taken.

### Data collection instrument

An open-ended thematic topic guide was designed and used to conduct IDIs. The guide covered several topics, including awareness and knowledge about sexual, reproductive and maternal health rights, access to healthcare services, and specific sexual, reproductive and maternal healthcare needs of women with different disabilities. Generally, the guide captured both positive and negative childbearing motivations. Positive motivations disposed WWDs toward having a child, while negative motivations disposed them toward avoiding childbearing [29]. Specific questions that were asked in relation to desire and motivation for childbearing included: “Is it important to have your own child?”; “Do you want to have your own child, and why and why not?”; “Why did you have a child?”; “Did you plan to have a child or it was unplanned?”; “Looking into the future, do you want to have a (or: another) child of your own?”; “How many children would you like altogether, and why?”; “If it were possible, would you want to have a child of your own; How many and why?” The questions were often adjusted to address the specific needs of participants with different impairments.

The guide was first developed in English. Our four research assistants translated it into the five local dialects. An independent language specialist (one per dialect) then checked the quality of the translation. All corrections were made before the guide was pretested. Following the pretest, further corrections were made before a final guide was agreed upon and used to collect data.

### Data processing and analysis

Thematic content analysis approach was used to analyse the data. This involved several steps. To begin, independent language translation specialists were engaged to transcribe all audio recorded interviews in the original interview language (i.e. *Gonja, Twi, Dagbaani, Moar*, *Komba* or English). All non-English transcripts were translated into English. The sign language expert also transcribed the sign language interviews into English. To ensure transcription quality, the first author and all the research assistants performed back-to-back translations on selected transcripts. All errors were corrected. All transcripts were edited to correct grammatical mistakes without altering original meanings. We read the edited transcripts severally to gain general understanding of the data. All transcripts were imported into QSR NVivo 11 software for data coding. The coding process involved critical review of each transcript, followed by coding into emerging themes. Finally, the themes were presented, and relevant quotes from the transcripts were used to support identified themes.

### Quality assurance and analytic rigour

In order to ensure data quality and analytic rigour, a number of measures were implemented. Firstly, our research assistants were trained on several aspects of the research and data collection tools, including explaining the main objective of the study and the data collection technique. Secondly, the interview guide was pre-tested, which helped us to reframe unclear questions. Thirdly, the PI (first author) was actively involved in supervising the research assistants during the data collection process to ensure data quality. Finally, we regularly held meetings with research assistants to review the data collection process as well as reflect on how our individual biases could affect the data. This process of continuous reflexivity during the data collection process ensured that questions were appropriately asked and that our personal biases were minimised.

## Findings

### Participants’ characteristics

The youngest participant was aged 18 years and the oldest was 47 years. A little over half of the participants (39) resided in rural areas, and the rest came from urban or peri-urban areas. Most of the participants (58) had no formal education. About half of the participants (37) have never married; 15 were currently married; five were cohabiting; another 15 were either divorced or separated; and five were widowed. Only 11 of the participants worked for monthly pay – nine in public sector, and two in private sector. Majority of the participants (48) had at least one child. Nine participants had no children but were pregnant at the time of the research; 15 participants said they believed they were fertile but did not yet have a child and were also not pregnant; and five participants said they were infertile either because of unknown reasons or due to a doctor’s advice not to get pregnant.

### Desire for children

The first major theme that emerged relates to desire to have children. Except four participants, all expressed great desire to have their own children:

*“As you can see, I already have two children; I have always wanted to have children, so I am happy that I have them.”* (Speech Impaired, Savelugu-Nanton).

“*I have not given birth yet, but I certainly want to have children; why will I not want to have them, except that Allah does not will it?”* (Visually impaired, Central Gonja).

Even those participants who said they were infertile reported how unhappy they felt on a daily basis about not being able to have children.

*“My parents gave birth to only female children, so when I was growing up I always wanted to have a brother but I did not have one … so I said that I must give birth to a baby boy so that he can compensate for my loss. Well, I did not know that this disability will happen and I will not be able to have my own children. I really would love to have my own children, even if it is one … and especially a boy, but now I cannot, and that pains me.”* (Physically impaired, Central Gonja).

When adoption was raised as an option, only one out of the five participants who said they were infertile was supportive of the idea. Several reasons were given to explain why adoption may be practically impossible or socially undesirable:

*“Yes, I know adoption is an option, but do you think orphanages or anyone will easily give out children for adoption by a woman with my condition? I do not think so … anytime I ask any of my friends or family members to let their children come and stay with me and help me, they often refuse … you know there is stigma against people like myself.”* (Woman suffering epilepsy, Central Gonja).

“*Like you asked about adoption, that could be a solution; but you know in our community if you adopt, people will talk … gossip that you cannot have your own child; that is why you have gone to adopt somebody’s child. I am sure my husband will not even accept it; he might say, ok then, I will leave you [divorce me] and go and marry a woman who can have children. So, that is why I do not think this adoption thing will work.”* (Visually impaired, Savelugu-Nanton).

In terms of the number of children they desired to have, many reported that they desired as many children as God/Allah would give them. This view was particularly pronounced among participants from rural settings:

*“Children are a gift from Allah, so I will have as many as Allah permits.”* (Speech impaired, Bunkpurugu-Yunyoo).

“*I already have one, and as you can see I am currently pregnant … I will have as many children as God will give me … why will I not have children if God gives them to me?”* (Physically impaired, Central Gonja).

Others said that their desired number of children would depend on whether they would be able to support those children, including sending them to school. This perspective was widely expressed among participants from urban settings.

*“For me, the number of children I will have will depend on whether I can support them … right now my husband and I are not living bad … so maybe we can take care of three or four children … but who knows, our situation could change tomorrow.”* (Woman suffering albinism, Bunkpurugu-Yunyoo).

Among the four participants (three from urban settings and one from a rural area) who said they did not want to have children, two said they did not desire children because they did not think they will be able to take care of a child if they had one.

*“Even if my husband will support me, I think it will be too difficult to take care of a child given my circumstances. So, I really do not want to complicate things with a child.”* (Physically impaired, Bunkpurugu-Yunyoo).

“*I do not have anything against people with disabilities who have children … I just feel that in my situation I will not be able to take care of a child physically, emotionally and financially. Will people in this community not say I am irresponsible if I have a child that I cannot take care of? That is why I said I do not want a child.”* (Woman suffering eplipsy, Central Gonja).

The other two did not want to have a child because they wanted to focus on their careers:

*“I know by God’s grace if I want to marry and have a child I will be able to do it. I know I will also be able to take good care of the child; but I have chosen not to have a child because I want to focus on my career … go back to school … I want to study law and become a lawyer.” (*Physically impaired, Savelugu-Nanton)*.*

In sum, majority of our study participants, irrespective of disability type and place of residence, were very desirous of having their own children, the number of children desired being dependent on both nature (Allah/God), and economic capability.

### Motivations for childbearing

To fully understand why many participants expressed great desire to have their own children, we explored the underlying motivational factors for childbearing. Five main thematic motivational factors emerged. These included the joy of motherhood, childbearing as a means of challenging stigma and negative stereotype, social security, economic security, and self-actualisation. In the next sections, we present our thematic analysis with supporting quotes to illustrate WWDs’ motivations for childbearing.

#### The joy of motherhood

Nearly all participants (69) who expressed a desire to have their own children reported that the joy of being a mother was an important motivational factor.

*“What is more joyful than being a mother? For me, the joy I got when I became pregnant and successfully gave birth is the reason I will have another child.”* (Visually impaired, Savelugu-Nanton).

“*For me, the motivation is the joy of motherhood. Like we say in this community, motherhood is like a bitter-sweet drink … it is difficult but there is nothing sweeter than having your own children, being able to play with them and call them your own”* (Physically impaired, Savelugu-Nanton).

Many of these participants agreed that motherhood comes with responsibility but noted that even if the children one bears do not grow up to become important persons in society, the experience of motherhood, in and of itself, is a very valuable and joyful one.

#### Challenging stigma and negative stereotype

Many participants reported that they were motivated to get pregnant and bear their own children because of negative stereotypes people in their communities held about PWDs when it comes to sexuality and child bearing.

*“For me getting married and getting pregnant and giving birth to my own children is a way of telling people and society that yes, I have a severe disability but I am not asexual … I can have sex, get pregnant and give birth … it is a way to say me too I can do it.”* (Physically impaired, Savelugu-Nanton).

“*The reason I want to have many children is because of society … if you have a disability like me, people in this community think that you should only be concerned about your disability and not matters of sex, marriage or childbirth. Some even think that because of my disability, I cannot care for a child. So, because of all these negative things, I want to disprove all those who think I cannot do it”* (Visually impaired, Central Gonja).

One participant also narrated her experience like this:

*“When I was growing up and did not have a disability, I used to tell my mother and fellow girls about how I want to marry a very handsome man and give birth to beautiful children. But when my disability happened, everything started to change … anytime I talked about marriage or childbearing, my mother will just say I should concentrate on my disability and stop disturbing her. As for the girls, they didn’t even come around often, and when they did, they only talked about my disability … anytime I want to talk of marriage or having children, they just laugh and ask me to concentrate on my disability. So, because of this, I said I was going to have a child, and now I have this strong boy here.”* (Physically impaired, Bunkpurugu-Yunyoo).

When asked about how childbearing enabled them to challenge existing stigma and stereotypes, many participants indicated that their experience of disability gave them opportunities to even educate their children and potentially the larger society about difference.

#### Children as social security

Another factor that motivated childbearing among WWDs’ is social security. Participants talked about social security in three distinct but inter-related ways. Many participants, especially those in marital relationships, talked about childbearing as a means to socially secure their position in marriage:

*“My motivation is that in our society, people expect us [referring to women], especially if you are married to have children. So, if you are married and you do not have children, then it is a big issue … you may lose your marriage...but now that I have children, at least I know even if my husband leaves me [referring to divorce], it will be for something else. So, you can say that some of us give birth to secure our marriage and position in society.”* (Speech impaired, Bunkpurugu-Yunyoo)*.*

The issue of securing marital position was particularly pronounced among Muslims where polygyny was common.

*“Are you asking why I gave birth … I am married … and I am expected to give birth. Everyone, especially my husband, expects me to give birth. I am sure if I did not, by now I will be out of my husband’s house. The other thing is that, if I do not and my two other co-wives give birth, do you think my husband will remain married to me? I can tell you that if I did not have two sons for my husband, he would have chased me out of his house long ago. But now because of these children, he respects me despite the fact that I have this disability.”* (Woman suffering albinism, Savelugu-Nanton).

Apart from securing marital unions, several participants also described how childbearing has brought them or could bring them social respectability, thereby protecting them from violence and abuse:

*“You are asking why I had children? Who will defend me if not my children? As a person with a disability, people used not to respect me … I suffered abuse all the time. But when I had my children and as they grew older and were successful [one is a teacher, one is a nurse, and two are now in school], people started to respect me. Besides, people know that if they abuse me and my children hear about it, there will be trouble. So, I can say that I am doing well today and people in this community respect me because of my children, and that is why if I were younger, I would probably have more children.”* (Visually impaired, Central Gonja).

In this way, childbearing acted as a social compensator for their disability, thereby increasing their acceptability and social respectability. Others also said that they were motivated to have children because it was the only way to ensure perpetuity of their own lineage and prevent generational loss.

*“I am the only child of my parents; if I do not have children, then our lineage will just die out in the future. Besides, if I marry and do not have children, then my husband’s lineage will also collapse. I do not want to be the one responsible for collapsing my parents’ or husband’s lineage … that is bad omen, and that is why it is important for me to have children no matter how hard it is to care for them.”* (Physically imapired, Bunkpurugu-Yunyoo)*.*

#### Economic security

Participants’ motivations for childbearing were also related to economic security, especially in the future.

*“Now that my husband and I are still a little stronger, life is very hard … no money and regular food. You can imagine what will happen in the future if there is no one to provide for us. So, my children, they are my future.”* (Physically impaired, Savelugu-Nanton).

“*For me, I want to give birth … I want a boy … somebody who will provide my needs when I am old.”* (Hearing Impaired, Central Gonja).

Participants who were physically or visually impaired particularly talked about how by having children, they have been able to engage in productive economic activities, which contributed not just to improving their current economic situation, but also their future economic security via savings:

*“Before I started having children, I could not do much … my movement was very restricted …I could not go to the farm to harvest crops. But when I started having my children, they helped me …they became my eyes …they led me to the farm and even to the market. This has really helped me and I believe it will help me in the future. But for the fact that I had my own children, I do not know what would have happened to me especially after the death of my husband”* (Visually impaired, Bunkpurugu-Yunyoo).

Indeed, several accounts across different disability types and spatial contexts highlighted the economic vulnerability of WWDs, and how childbearing presented an opportunity to improve their future economic security.

#### Self-actualisation

Another motivation for childbearing among a substantial number of participants (48) is self-actualisation. Participants talked about self-actualisation in two ways. One was in relation to women’s biological role as carriers of human life:

*“As women, one of our key roles is to be able to get pregnant and successfully carry a pregnancy to term and give birth. So, if I am able to do this, then I think I have fulfilled my role. It makes me feel self-actualised, and this is why I want to have a child.”* (Physically impaired, Bunkpurugu-yunyoo)*.*

Others also talked about self-actualisation in terms of societal expectation of women not only to bear children and become mothers, but also to be able to nurture their children into successful citizens.

*“I really cannot fully explain the sense of accomplishment I felt after giving birth to children who are now making impact in society. I feel very fulfilled and accomplished that with all my disability, I have been able to bear children who are now playing important roles in our community and in the country. This is a motivation to every woman, including WWDs, to have children.”* (Hearing and speech impaired, Savelugu-Nanton)*.*

Participants who were already mothers repeatedly talked about their sense of empowerment through their motherhood experience, and how becoming involved in caring and intimate relationship with their children was very fulfilling.

*“I think one reason why I want to have one or two more children is this sense of fulfilment I get from my children. You know in our society, people expect women to give birth and raise their children to be responsible citizens. Once you do that, then you would have played an important role as a mother…right now, when I look at my first son, I feel like he has really made me fulfilled even though he is still young…he is making me proud…he finished his basic school certificate examination with flying colours…I am told he is the best, and now everybody in this community is praising us. He is also doing well in church…he is a mass/church servant, and people say he is responsible. This brings me fulfilment, and this is why I want more children.”* (Visually impaired, Central Gonja).

## Discussion

This study is one of the first in Ghana to focus on exploring childbearing desires and motivations among WWDs. Several aspects of our findings deserve further discussion and reflection. Following Miller’s [28, 29] conceptual discussion on why people have children, this study has highlighted the experience of the joy of motherhood as an important driving factor in WWDs’ desire to have children. This is very consistent with a number of recent studies that showed that many WWDs’ experienced motherhood as a very joyful event. For instance, Walsh-Gallagher and colleagues’ study showed that WWDs perceived the experience of pregnancy and motherhood as one to celebrate, considered pregnancy an achievement, and felt that pregnancy resulted in a bonding experience and confidence in their ability to achieve a life goal [46]. One qualitative study from Ethiopia also reported that WWDs expressed their feelings of motherhood with a good spirit, with many considering motherhood as a source of joy [27].

Our finding that many WWDs valued the experience of being mothers provides important counterpoints to existing societal prejudices and misconceptions that often portray disabled women as incapable of desiring, having, caring for, and enjoying children [47]. Our findings are however in consonance with contemporary scholarship around reproductive justice, which acknowledges the right of all women and men to have or not have children as well as how the experiences of childbearing could be empowering for individual women and men [48]. Our results clearly showed that though many WWDs acknowledged the difficulties that motherhood could bring, they felt the experience of being a mother, including the benefits of bonding with children, is both joyful and healing.

Our findings also revealed that many WWDs are motivated to have children in order to proof that they are both biologically and socially capable of being mothers. As noted throughout this paper, the mothering experiences of many WWDs are fraught with barriers, stigma, and surveillance. Our findings in this paper generally demonstrate that many WWDs are acutely aware of these prejudices and barriers, and are actively negotiating and challenging existing stereotypes by getting pregnant and bearing their own children. This agency clearly has theoretical and practical implications. First, it calls for critical engagement with disability in a way that questions how disability has come to be primarily conceptualized as a deficit or negative outcome in the first place [47]. Second, this agency demonstrates that many WWDs have successfully resisted internalizing stigmatizing discourses about disability, sexuality and motherhood. In this regard, community-based public education on issues of disability, sexuality and motherhood are required to improve public understanding around disability issues, change negative societal attitudes about disability and motherhood, and reduce stigma.

Several of our participants also reported fulfilment, empowerment and self-actualization as important motivations for childbearing. For many of the WWDs in our study, pregnancy and childbearing reaffirmed their feminine identity and sexuality as well as their self-worth as women. In previous studies, WWDs have commented on how becoming pregnant was experienced as an important achievement and affirmation of femininity, and how the experience of pregnancy confirmed that their biological bodies could function like those of women without disabilities [49]. Our findings particularly showed that being a mother and doing mothering were fulfilling life events for many WWDs - events that caused them (and others) to realize their abilities rather than disabilities [46, 49]. As the pronatalist orientation of our research context have tended to encourage childbearing among women as a marker of biological and social accomplishments, we are not surprised that many of our participants viewed childbearing as a means to self-actualisation. Indeed, within the context of Miller’s conceptual framework that guided this study, pronatalism and self-actualisation are, respectively, community and individual level factors that strongly stir up disabled women’s desire for childbearing. This said, we think it is important that WWDs not only see their self-worth through childbearing. Expanding access to education and increasing training and skills development opportunities for young girls with disabilities could potentially allow them to participate and contribute meaningfully to other facets of their social and community lives.

Fear of social and economic insecurities also emerged as important motivational factors for childbearing. Indeed, demographers and economists have historically found demand for labour and future economic insecurity as reasons underlying childbearing desires and intentions among men and women without disabilities [28–30]. Within disability studies, recent studies have similarly reported that many WWDs perceived having children as an investment for the future in terms of social support and labour provided by grown children [49]. In this study, many WWDs across different spatial contexts highlighted their economic vulnerability, and how childbearing may be the only way to achieve social acceptance and respectability as well as economic security. Given that many WWDs linked their precarious social and economic situations to childbearing, it is essential that social and economic development policies and programmes in contexts like Ghana target and benefit WWDs. Mainstreaming disability issues into local social and economic development could potentially improve the wellbeing of WWDs as well as improve sexual and reproductive health outcomes, including better planning and timing of childbirth.

Finally, a few of our participants believed they were fertile but indicated that they did not want to have children. Reasons for the lack of desire for children ranged from practicable challenges of caring for children, voluntary infertility to fears that they may give birth to children with disabilities. We think it is very progressive that some WWDs are making childbearing decisions based on careful reflection on their social, economic and career situations. However, we are concerned that some of these decisions appear to be influenced by internalized stigma and fear of disability transmission. Not only did some WWDs wanted to avoid having a child with a disability but also, they seemed to endorse a belief that WWDs should not have children. We think reproductive health researchers and practitioners are well situated to provide support for WWDs who desire pregnancy and motherhood but may be facing uncertainty about risks and benefits of having a child with a disability.

Taken together, the conceptual framework we followed has enabled us to explore and reveal the formation of childbearing desires and underlying motivational factors among WWDs. These findings have implications for theory, reproductive health policy, and service delivery. First, our finding that WWDs are sexually active and are having children or desire to have children challenges existing mis/perceptions about the asexuality and motherhood incapability of WWDs. Second, these findings suggest a need to adequately attend to the sexual and reproductive healthcare needs and challenges of WWDs both in reproductive health policy-making and service delivery. In calling for a focus on the sexual and reproductive health needs of WWDs, we take cognizance of the fact that several of our research participants wanted to have as many children as God/Allah wills it. The rights of WWDs to marry and found a family and retain their fertility (Article 23), and have access to sexual and reproductive healthcare (Article 25), are all guaranteed under the UN Convention on the Rights of Persons with Disabilities [2]. Indeed, the right to have or not have a child is an important principle in contemporary discussions around reproductive justice [48], and we fully support the right of WWDs to have their desired number of children and to parent their children in safe and healthy environments. We are however concerned about the possible adverse reproductive health outcomes that unfettered childbearing could bring to individual WWDs and their families. For instance, there is evidence to show that as the number of births for any particular women increases, the risk of maternal morbidity and mortality also increases [50]. Also, given the context of our research where social and family support for WWDs is often limited, the daily practical social and economic challenges of raising many children is a concern. There is therefore a need to target WWDs with modern sexual and reproductive health services (e.g. modern contraception and family planning) and information to ensure that their childbearing decisions are made in an informed and empowered manner. In addition, we suggest mainstreaming of disability issues into reproductive health policies and programmes to address misguided public perceptions and assumptions that perpetuate stigma against WWDs, and potentially reduce medical, social and psychological support for WWDs who desire to be mothers. Finally, the agency demonstrated by WWDs in this study in relation to their reproductive choices has implications for reproductive health policy and service delivery in terms of a need to consider and provide quality sexual and reproductive healthcare information and services to address the specific reproductive health needs of WWDs. We call for increased sexual and reproductive health education for WWDs who desire to be mothers. This education could help empower them to make informed sexual and reproductive health decisions with beneficial and dignified outcomes.

The findings reported in this paper should be interpreted with a number of limitations in mind. First, our sample comprised only 77 participants. While this sample size was adequate for a qualitative study like ours, we acknowledge the limitations of generalizing our results beyond our study context. This is particularly so given the pronatalist and patriarchal orientation of our research context. Secondly, while we implemented a number of research and data quality assurance measures, we acknowledge that some meaning may have been lost in the transcription and translation of non-English interviews. These limitations notwithstanding, we believe our findings offer important pointers to the need to adequately provide for, and address, the sexual and reproductive aspirations, needs and challenges of WWDs in policy and practice. Finally, disability often intersects with other vulnerabilities such as poverty and gender-based violence. While we endeavoured to include women with different disabilities in our study, our analysis did not explore in detail how the type of disability influences motivations for childbearing. Similarly, our study has not explored the intersections between disability and other vulnerabilities and how this intersectionality impacts on motivations for childbearing among women with different disabilities.

## Conclusion

Contrary to popular perceptions about WWDs’ asexuality or lack of desire for children, our study showed that many WWDs are sexually active, desired to have children, and intended to have as many children as they could support. These findings challenge existing negative public perceptions and stereotypes about the status of WWDs in relation to sexuality, childbearing and motherhood. More importantly, our findings suggest that if the Sustainable Development Goals (SDGs) related to universal access to sexual and reproductive healthcare are to be attained, WWDs must be targeted with quality sexual and reproductive healthcare information and services, including family planning information and services. If local governments and the global health community are truly interested in improving health for all, now is an appropriate time to question discourses around disability and its relationship to sexuality and motherhood. We should also actively identify and explore our own spheres of ignorance, and as well acknowledge that there are aspects of the experiences of WWDs that we know little or nothing about. We should also then be open to the possibility that these experiences may provide valuable lessons to propel progress towards achieving the SDGs related to universal sexual and reproductive healthcare. In this regard, we believe reproductive health practitioners are well situated to provide counselling and support for all WWDs who desire pregnancy and motherhood. Funding agencies and researchers also have critical roles to play in terms of directing research and deepening understanding of the reproductive health needs and challenges of WWDs, the context-specific strategies needed to develop and facilitate recognition of the sexuality and reproductive rights of WWDs, as well as how appropriate sexual and reproductive healthcare services could be made available and more accessible to WWDs.

## Data Availability

All relevant data are included in this paper.

## Abbreviations

CBSVs: Community-based Surveillance Volunteers
GFD: Ghana Federation of Disability Organisations
IDIs: In-depth Interviews
PWDs: Persons with Disabilities
SDGs.: Sustainable Development Goals
TFR: Total Fertility Rate
UNCRPWDs: United Nations’ Convention on the Rights of Persons with Disabilities
WWDs: Women with disabilities
